# Apigenin Restricts FMDV Infection and Inhibits Viral IRES Driven Translational Activity

**DOI:** 10.3390/v7041613

**Published:** 2015-03-31

**Authors:** Suhong Qian, Wenchun Fan, Ping Qian, Dong Zhang, Yurong Wei, Huanchun Chen, Xiangmin Li

**Affiliations:** State Key Laboratory of Agricultural Microbiology, Laboratory of Animal Virology, College of Veterinary Medicine, Huazhong Agricultural University, Wuhan 430070, Hubei, China; E-Mails: shqianhzau@163.com (S.Q.); iwenfan@163.com (W.F.); qianp@mail.hzau.edu.cn (P.Q.); sd4190130@126.com (D.Z.); abc911424@126.com (Y.W.); chenhch@mail.hzau.edu.cn (H.C.)

**Keywords:** foot-and-mouth disease virus, Apigenin, Antiviral, Internal ribosome entry site

## Abstract

Foot-and-mouth disease (FMD) is a highly contagious disease of domestic and wild ruminants that is caused by FMD virus (FMDV). FMD outbreaks have occurred in livestock-containing regions worldwide. Apigenin, which is a flavonoid naturally existing in plant, possesses various pharmacological effects, including anti-inflammatory, anticancer, antioxidant and antiviral activities. Results show that apigenin can inhibit FMDV-mediated cytopathogenic effect and FMDV replication *in vitro*. Further studies demonstrate the following: (i) apigenin inhibits FMDV infection at the viral post-entry stage; (ii) apigenin does not exhibit direct extracellular virucidal activity; and (iii) apigenin interferes with the translational activity of FMDV driven by internal ribosome entry site. Studies on applying apigein *in vivo* are required for drug development and further identification of potential drug targets against FDMV infection.

## 1. Introduction

Foot-and-mouth disease (FMD) is an economically devastating viral disease of domestic and wild cloven-hoofed animals, which is highly contagious and clinically acute. Outbreaks have occurred in every livestock-containing region worldwide. FMD is caused by FMD virus (FMDV), which belongs to the *Aphthovirus* genus of the *Picornaviridae* family and contains a single-stranded positive RNA genome of about 8500 bases surrounded by four structural proteins to form an icosahedral capsid [1]. Seven serotypes (A, O, C, Asia 1, and South African Territories 1, 2 and 3) have been identified on the basis of VP1 coding region sequence, and multiple subtypes occur in each serotype [2,3,4]. An internal ribosome entry site (IRES) element has been identified in the 5'-untranslated region (UTR) of the FMDV genome [5,6]. The FMDV IRES interacts with the L^pro^-generated C-terminal eIF4G cleavage product to drive FMDV RNA translation [7,8,9,10]. In addition to being a translation driver, FMDV IRES is considered as a virulence factor [11].

Currently, FMD control measures include animal movement restriction, infected animals slaughter, disinfection and vaccination program development [1]. Both the conventional inactivated vaccine and the Ad5-vector FMDV subunit vaccine can induce complete protection for seven days [12,13,14]. In addition, interferons (IFNs) work as the first line of host cell defense against viral infection [15,16]. IFNs can rapidly induce a nonspecific protective response against viral infection [17,18,19]. Some antiviral drugs that target viral proteins, such as LprO, have been developed to treat FMD [20,21,22]. It was reported that 1,2,4,5-Tetrahydro-[1,4]-thiazepino-[4,5,a]-benzimidazole inhibits FMDV infection through interaction with FMDV 2C protein [23].

Apigenin, a member of the flavone family, is a nontoxic and nonmutagenic dietary flavonoid found in parsley, artichoke, basil, celery, and other plants [24]. Apigenin possesses various pharmacological effects, such as anti-inflammatory, anticancer, antioxidative and antiviral function [25,26,27,28,29]. This study, investigates the antiviral effect of apigenin on FMDV infection *in vitro* and further explores apigenin inhibits the FMDV infection through suppressing the FMDV IRES activity.

## 2. Materials and Methods

### 2.1. Compound and Antibodies

Apigenin and baicalein were obtained from Sigma-Aldrich (St. Luis, MO, USA). Chrysin, liquiritigenin, quercetin, kaempferol, and galangin were purchased from SHANGHAI YUANYE BIOLOGICAL TECHNOLOGY CO., LTD. Mouse anti-GFP monoclonal antibody (mAb) was purchased from Proteintech, China. Mouse anti-β-actin mAb was obtained from ABclonal Biotech Co., Ltd, Cambridge, MA, USA. Rabbit anti FDMV (O/ES/2001) VP1antibody was generated in our lab.

### 2.2. Cells and Viruses

Our antiviral studies *in vitro* were performed on BHK-21 cells. BHK-21 cells were maintained in Dulbecco’s Modified Eagle Medium (DMEM; Gibco, Grand Island, NY, USA) containing 10% fetal bovine serum (FBS; Gibco, Grand Island, NY, USA) and cultured at 37 °C in a humidified 5% CO_2_ incubator. Type O FMDV strain O/ES/2001 was propagated on BHK-21 cells and titrated by plaque forming unit (PFU) assay [30].

### 2.3. Plasmids Construction

The overlap PCR method was used to generate IRES-fused-GFP fragment. The primer pairs are listed in Table 1. The IRES-fused-GFP fragment was then sub-cloned into pEGFP-C1 (Clontech) vector at the restriction sites *of NheI* and *XhoI* to result in pIRES-GFP.

**Table 1:** Primers used in this study.

Primer	Sequence 5'→3'
IRES-GFP-F	GGGAATTCCCACAACTGAGAAAACTCG
IRES-GFP-R	CCTCGAGCTACTTGTACAGCTCGTCC
Over-R	CGCCCTTGCTCACCATGGATTTAGT
Over-F	ATGGTGAGCAAGGGCGAGGAGCTGT
GFP-F	GCAGAAGAACGGCATCAAGG
GFP-R	GTGCTCAGGTAGTGGTTGTC
GAPDH-F	TCATGACCACAGTCCATGCC
GAPDH-R	GGATGACCTTGCCCACAGCC
3D-F	GAACACATTCTTTACACCAGGAT
3D-R	CATATCTTTGCCAATCAACATCAG

### 2.4. Cytotoxicity Assay

BHK-21 cells were seeded at a density of 10^5^ cells into 96-well plate and incubated for overnight. The medium was added with different concentrations of apigenin from 0 to 160 µg/mL. The cells were mock treated with DMSO. Both treated cells and mock treated cells were incubated for 48 h. The 50% cytotoxicity concentration (CC_50_) of apigenin was detected using CellTiter 96^®^ AQueous One Solution Cell Proliferation Assay Kit (Promega, Beijing, China).

### 2.5. Infection and Antiviral Effects Assay

The antiviral activity of apigenin *in vitro* was determined in 96-well plates or 24-well plates. BHK-21 cells were infected with FMDV at an multiplicity of infection (MOI) of 0.1 per well. Cells and virus were incubated in 37 °C for 1 h, and then washed three times with phosphate-buffered saline (PBS) to remove the virus. After that, mediums containing different concentrations of apigenin were added into cells. At 24 h post-infection (hpi), cytopathic effect (CPE) induced by the FMDV was observed. The titer of progeny virus and expression of FMDV VP1 were determined to measure the antiviral effect of apigenin.

### 2.6. Time-of-Addition Assay

In time of drug-addition assay, BHK-21 cells were seeded in 24-well plates and infected with FMDV at a MOI of 0.1. Apigenin at 20 µg/mL was added at time points respectively representing that of viral adsorption (−1 h), during adsorption (0 h), or post cells entry (+1 h) [31]. The inhibition rate was evaluated at 24 hpi.

### 2.7. Titration of Virus

Virus titers were determined using PFU assay [30]. Briefly, BHK-21 cells were seeded in 24-well plates 24 h prior to infection with 10-fold serially diluted FMDV strain O/ES/2001 samples. After 1-h incubation in 37 °C, un-adsorbed viruses were removed. After washing three times with PBS, cells were overlaid with 2% methylcellulose and incubated at 37 °C for 2 d. Finally, the cells were fixed with 10% formaldehyde and were stained with crystal violet. The number of plaques was observed and statistically analyzed.

### 2.8. Western Blotting Assay

Cells were treated with lysis buffer containing 1.19% HEPES, 0.88% NaCl, 0.04% EDTA, 1% NP40, and protease inhibitor (Roche). The protein concentration of cell lysates was determined with bicinchoninic acid protein assay kit (Pierce, Meridian Rd, Rockford, IL, USA). Equal amounts of protein were separated using 12% sodium dodecyl sulfate–polyacrylamide gel electrophoresis (SDS-PAGE) and transferred to polyvinylidene difluoride (PVDF) Western blotting membranes (Roche). The membrane was blocked with 5% nonfat milk in Tris buffered saline (TBS) and incubated with specific antibodies. The expression of β-actin was detected with an anti-β-actin mouse monoclonal antibody (Beyotime, Shanghai, China) to demonstrate the equal loading of protein samples.

### 2.9. Semi-Quantitative PCR and Quantitative Real-Time PCR

Total RNA was extracted using TRIzol reagent (Life Technologies, Carlsbad, CA, USA) according to manufacturer’s instructions. Reverse transcription was performed with 1 μg of total RNA using the First Strand cDNA Synthesis Kit (TOYOBO, Kita-ku Osaka, Japan) according to manufacturer’s instructions. The cDNA level of FMDV 3D gene was determined by relative quantitative real-time PCR through using SYBR Green Real-time PCR Master Mix (TOYOBO, Kita-ku Osaka, Japan), and fluorescent signals were analyzed by an ABI StepOne Plus system (Applied Biosystems, Foster city, CA, USA). The cDNA level of GFP gene was analyzed using semi-quantitative PCR. PCR products were visualized by electrophoresis in 2% agarose gels stained with ethidium bromide. GAPDH was used as the internal control. Primer pairs are shown in Table 1.

### 2.10. Statistics

GraphPad Prism software Version 5 (GraphPad Prism Version 5, GraphPad Software, La Jolla, CA, USA, 2012) was used in this study. All experiments were performed at least three times with reproducible results. Statistical analysis was performed using two-tailed student t-test. Statistical significance: * *p* < 0.05, ** *p* < 0.01, *** *p* < 0.001.

## 3. Results

### 3.1. Apigenin Inhibits FMDV Infection

**Figure 1:**
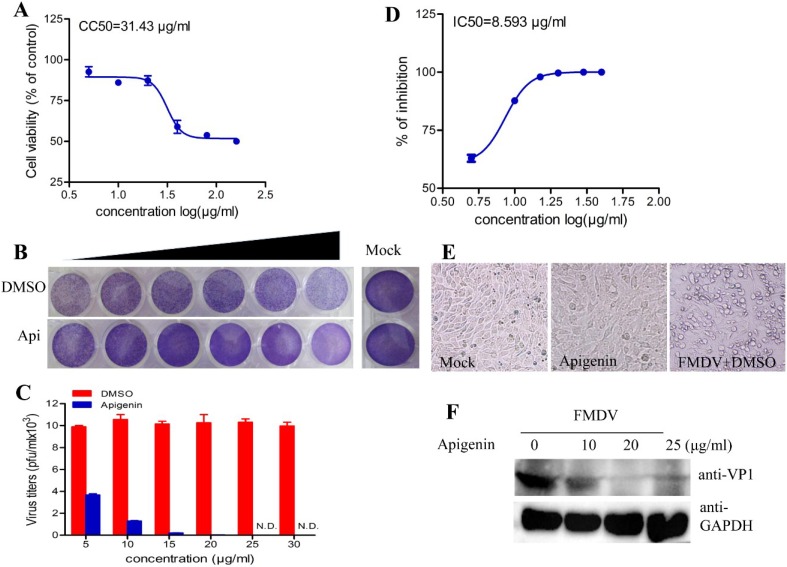
Apigenin-induced inhibition of FMDV infection. (**A**) Cytotoxicity of apigenin in BHK-21 cells. BHK-21 cells in 96-well plates were treated with apigenin at specific concentrations. Cell viability was assayed at 24 h post-treatment using cell proliferation assay. The absorbance of OD490 was analyzed in triplicate samples, and GraphPad Prism Version 5 was used to extrapolate the CC_50_ values; (**B**,**D**) Determination of IC_50_ of apigenin anti-FMDV activity. BHK-21 cells in 96-well plates were infected with FMDV at an MOI of 0.1. At 1 h post adsorption at 37 °C, the cells were cultured in the presence or absence of apigenin at specific concentrations. The supernatants were harvested at 24 hpi. Progeny virus titers were determined via plaque forming unit assay; (**B**) Observations of FMDV plaques; (**C**) data statistics; and (**D**) IC_50_ were extrapolated using GraphPad Prism Version 5; (**E**) Apigenin inhibied FMDV-induced CPE. BHK-21 cells were mock-infected or infected with FMDV at an MOI of 0.1 in the presence of apigenin at a concentration of 20 µg/mL. Images were obtained at 24 hpi; (**F**) Apigenin inhibited FMDV VP1 expression. BHK-21 cells were infected with FMDV at an MOI of 0.1 in the presence of apigenin at concentrations of 10 µg/mL, 20 µg/mL and 25 µg/mL. VP1 expression was detected via Western blot at 20 hpi. GAPDH protein served as the loading control. These experiments were performed three times and the representative results are shown.

An infection assay in BHK-21 cells was conducted through cytopathic effect (CPE) observation and virus titer determination to assess the effects of seven flavonoids *i.e.*, apigenin, chrysin, baicalein, liquiritigenin, quercetin, kaempferol, and galangin on FMDV replication and infection. Both kaempferol and galangin at 10 µg/mL exhibited acute cytotoxicity. Only apigenin exhibited inhibitory action on FMDV infection (Figure S1). Thus, the antiviral activity of apigenin on FMDV infection was further studied. The CC_50_ value of apigenin was 31.43 µg/mL in BHK-21 cells (Figure 1A). Therefore, apigenin at different concentrations of 5, 10, 15, 20, 25, and 30 µg/mL was used for antiviral effect assay. BHK-21 cells in 96-well plates were infected with FMDV at a multiplicity of infection (MOI) of 0.1 and then treated with apigenin at specific concentration for 24 h. The results of plaque reduction assay revealed that the inhibitory effects of apigenin on FMDV replication were dose dependent. Apigenin at 20 µg/mL significantly suppressed infectious virion production, but the infectious virion was not detected at concentrations above 25 µg/mL (Figure 1C). The antiviral activity of apigenin against FMDV infection was measured by through IC_50_ determination. As shown in Figure 1D, the IC_50_ was evaluated to be 8.593 µg/mL.

FMDV infection could induce CPE in BHK-21 cells, but apigenin significantly inhibit FMDV-induced CPE at 20 µg/mL as shown in Figure 1E. Cells infected with FMDV but not treated with apigenin showed obvious FDMV-induced CPE, but those infected with FMDV and treated with apigenin did not exhibit any CPE. To further explore the antiviral activity of apigenin, the inhibition to FMDV production was also substantiated by measuring the reduction in VP1 protein expression. As shown in Figure 1F, VP1 expression was remarkably reduced in the samples treated with apigenin at 10 µg/mL. These results distinctly revealed that apigenin exerted antiviral activity against FMDV infection.

### 3.2. Apigenin Inhibits FMDV Infection during Post-Entry

**Figure 2:**
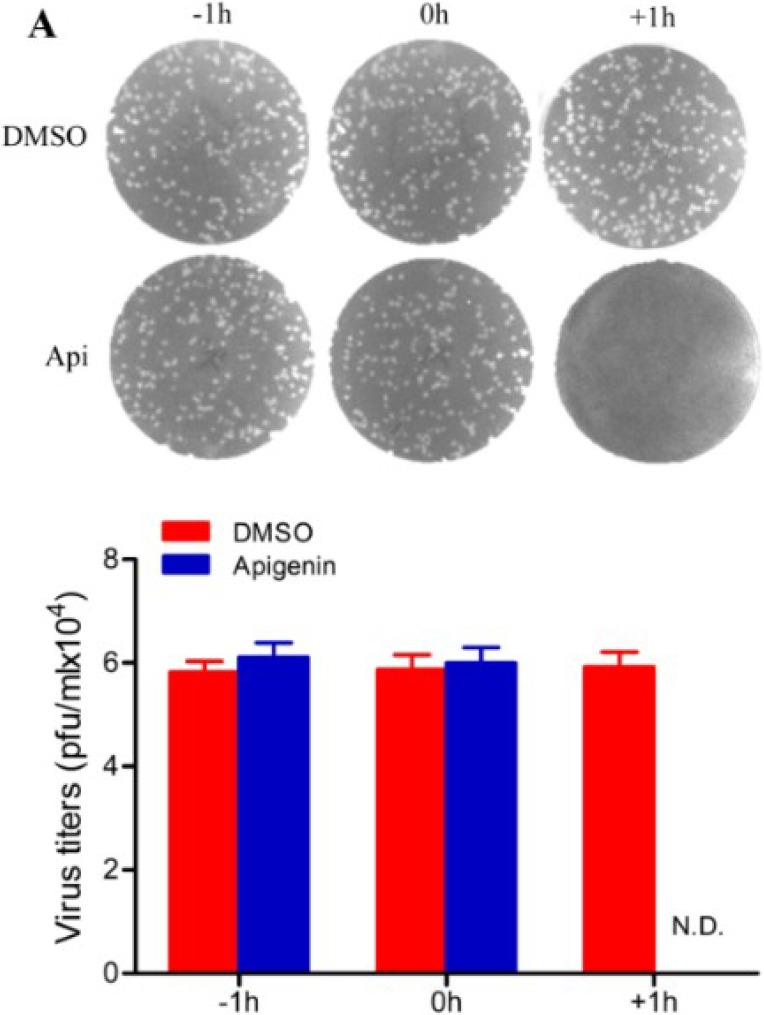
Time-of-drug-addition assay. BHK-21 cells were infected with FMDV at an MOI of 0.1 and then exposed to apigenin (20 µg/mL) at specific time points. −1 h: pre-infection 1 h. 0 h: during-infection. +1 h: post-infection 1 h. Apigenin exhibited a high inhibition rate at the post-infection stage. (**A**) Plaque reduction assay of apigenin; (**B**) FMDV viral titers. These experiments were performed three times, and the representative results are shown.

**Figure 3:**
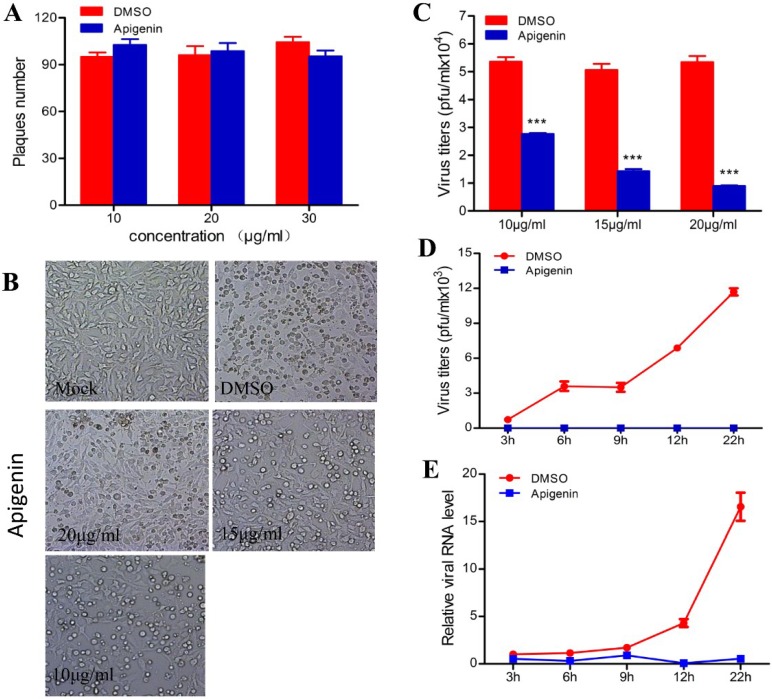
Apigenin has an intracellular antiviral activity. (**A**) Direct virucidal activity of apigenin against FMDV. Moreover, 10^5^ PFU FMDV particles were incubated with apigenin at specific concentrations for 2 h at 37 °C. BHK-21 cells were infected with the viral suspension that was diluted 1000-fold in triplicate to determine the direct virucidal activities of apigenin against extracellular FMDV; (**B**,**C**) Therapeutic effect of apigenin against FMDV infection. BHK-21 cells were infected with FMDV at an MOI of 0.2. Apigenin was added at specific concentrations at 12 hpi, and cells and cultural supernatants were harvested at another 12 hpi. (B) CPE images were obtained at 24 hpi; (**C**) Viral titers; (**D**,**E**) Apigenin inhibited FMDV intracellular replication. BHK-21 cells were infected with FMDV at an MOI of 0.1, and were cultured in the presence or absence of apigenin (20 µg/mL). Cells and cultural supernatants were harvested at specific time; (**D**) The progeny virus titers in the supernatants were determined via PFU assay; (**E**) The RNA level of the FMDV 3D gene was analyzed via qRT-PCR. GAPDH served as the normalizer. The experiment was performed three times independently.

As shown in Figure 2, a time-of-drug-addition assay was performed. Results showed that no inhibition of infection was detectable when apigenin was added at pre-infection and during-infection. However, FMDV infection was inhibited when apigenin was added at post-infection. The extracellular effects of apigenin against FMDV were determined by incubating the FMDV suspension using 10^5^ PFU with equal volumes of different apigenin concentrations for 2 h at 37 °C. Subsequently, BHK-21 cells were infected twice with the viral suspension that was diluted 1000-fold for plaque reduction assay [32]. As shown in Figure 3A, apigenin did not exhibit any significant direct extracellular antiviral activity on FMDV. The therapeutic effect of apigenin was investigated. BHK-21 cells were infected with FMDV at an MOI of 0.2. At 12 hpi, the FMDV-induced CPE was greater than 60%; meanwhile, different concentrations of apigenin were added and cultured for another 12 h. As shown in Figure 3B, FMDV-induced CPE was significantly attenuated during the progress of FMDV infection using apigenin. The infectious virion production was determined via plaque assay. The results are shown in Figure 3C, where progeny virus production was inhibited by apigenin in a dose-dependent manner. This result suggests that apigenin can restrict the progress of FMDV infection.

The intracellular antiviral activity of apigenin in FMDV infection was also investigated. FMDV-infected cells (MOI = 0.1) were treated or untreated with apigenin (20 µg/mL). Cells and culture supernatants were harvested at indicated times. The progeny infectious virion production in the supernatant was measured using PFU assay. The total RNA of cells was extracted to analyze the FMDV viral RNA level via qRT-PCR. As shown in Figure 3D, the rapid proliferation of FMDV started at 6 hpi and lasted until 22 hpi. However, no infectious virion was detected in the apigenin treated group. A consistent result was found in the viral RNA level analysis (Figure 3E). Therefore, these results suggest that apigenin inhibited FMDV infection by targeting post-cell entry events.

### 3.3. Apigenin Suppresses FMDV IRES Activity

**Figure 4:**
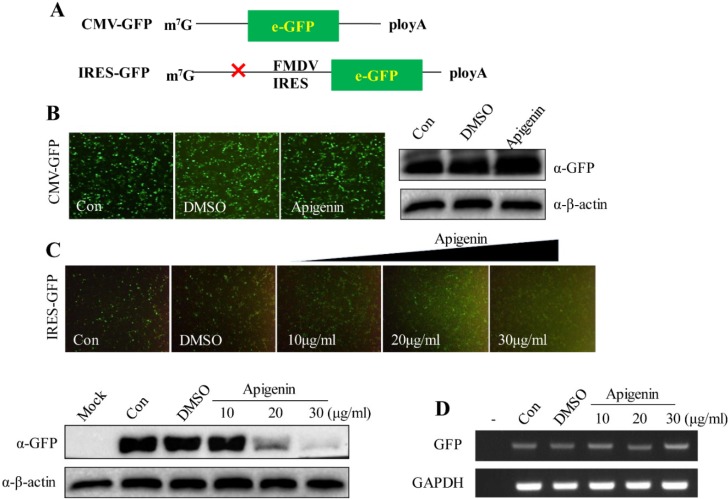
Apigenin exerts an inhibitory action on FMDV IRES activity. (**A**) Diagram of the FMDV IRES activity detection system. The eGFP fragment of pCMV-eGFP-C1 was replaced by IRES-GFP. The plasmid was named IRES-GFP; (**B**) Apigenin did not affect GFP expression mediated by a cap structure. BHK-21 cells were transfected with CMV-GFP plasmids (2 µg). Apigenin (20 µg/mL) was added at 12 hpt. The images were obtained under a fluorescence microscope in another 24 h post-treatment. The cells were harvested, and the GFP protein level was determined via Western blot. Beta-actin was used as the normalizer; (**C**) Apigenin suppressed FMDV IRES-driven translational activity. BHK-21 cells were transfected with IRES-GFP plasmids (2 µg). After 12 hpt, apigenin was added at specific concentrations. The images were obtained under a fluorescence microscope at another 24 h post-treatment. The cells were harvested, and the GFP protein level was determined via Western blot. Beta-actin was used as the normalizer; (**D**) Apigenin did not affect the transcriptional activity of IRES-GFP. Apigenin suppressed FMDV IRES-driven translational activity. BHK-21 cells were transfected with IRES-GFP plasmids (2 µg). After 12 hpt, apigenin was added at specific concentrations. After 24 h, the cells were harvested, and the total RNA was extracted. The level of GFP mRNA was analyzed via semi-quantitative PCR. GAPDH served as the intracellular control. These experiments were performed three times, and the representative results are shown.

When cells were infected with FMDV, FMDV LprO cleaved the host eIF4G to shutdown host cap-dependent translation. However, FMDV can use the C-terminal of cleaved eIF4G to drive FMDV RNA translation [6,7,8,9,10]. FMDV IRES element drives viral RNA translation. Hence, we assumed that whether apigenin could suppress FMDV IRES-mediated translational activity. As shown in Figure 4A, GFP reporter plasmids were constructed to determine whether or not FMDV IRES is a target of apigenin antiviral effects. Results showed that the protein expression of GFP in cells transfected with pEGFP-C1 was not affected by apigenin at 20 µg/mL (Figure 4B). However, the GFP protein level in the cells transfected with pIRES-GFP in the presence of different apigenin concentrations was suppressed in a dose-dependent manner (Figure 4C). To eliminate the suppressing effect of apigenin on the transcription of the GFP gene, the mRNA level of GFP was analyzed. The cells were transfected with pIRES-GFP plasmids and were treated or untreated with apigenin at different concentrations for 24 h. Then the cDNA level of GFP was analyzed through semi-quantitative PCR. As shown in Figure 4D, the transcription of the GFP gene was unaffected by apigenin. The results indicate that apigenin suppressed the IRES-mediated translational activity of FMDV instead of the transcription of GFP genes.

## 4. Discussion

As the etiologic agent of FMD, FMDV is a serious threat to the health and welfare of domestic and wild ruminants. FMD negatively affects the global trade of livestock products. During the OIE/FAO global conference on FMD in June 2009, 70 countries were officially recognized as FMD free. However, more than 100 countries were endemically or sporadically infected with FMDV.

Flavonoids are widely distributed in plants. Flavonoids are found in fruits, vegetables, nuts, seeds, stems, and flowers. Apparently, flavonoids possess various biological and pharmacological activities, such as anti-inflammatory [27,29,33], antimicrobial [34,35,36], anticancer [33] and antidiarrheal [37]. The molecular structure of the flavonoid compounds backbone is 2-phenyl-1,4-benzopyrone, which consists of two phenyl rings (A and B) and a heterocyclic ring (C) (Figure S2A). Flavonoids are classified into three types: flavonoids or bioflavonoids, isoflavonoids, and neoflavonoids (http://goldbook.iupac.org/F02424.html). This study, investigated the anti-FMDV activity *in vitro* of seven members of the flavonoid family: apigenin, chrysin, baicalein, liquiritigenin, quercetin, kaempferol, and galangin. The molecular structures of the seven flavonoids are shown in Figure S2B. These flavonoids were previously reported as antiviral agents for various viruses [26,28,32,38,39,40,41,42,43,44,45]. The present study found that only apigenin can exhibit antiviral effects on FMDV infection among the seven flavonoids (Figure S1).

Apigenin (4′,5,7-trihydroxyflavone) contains a hydroxyl group in its B-ring, and hydroxyl groups in its C-ring. Apigenin can be found in many plants and vegetables, including parsley, celery, celeriac, and chamomile. Apigenin exhibits various antiviral activities against numerous viruses *in vitro* and *in vivo*: enterovirus 71 (EV71) [26,28], hepatitis C virus (HCV) [41,46], Human Immunodeficiency Virus (HIV) [47], and adenoviruses [48]. Lv *et al*. reported that apigenin and luteolin differ in molecular structure by only one hydroxyl group in the B-ring. This difference confers apigenin and luteolin with different mechanisms to inhibit EV71 infection [26]. Apigenin can also inhibit EV71 IRES-mediated translation without a specific cell type. However, luteolin shows no significant inhibitory activity on EV71 IRES-mediated translation. The amount and site of hydroxyl groups in flavone backbone are essential for the antiviral activity of flavone. Meanwhile, Zhang and collaborators reported that apigenin disrupts viral RNA association with host transacting factors against EV71 infection [28]. Both of these studies demonstrated, through bicistronic reporter assay, that apigenin inhibits viral IRES-mediated translational activity. An IRES element in the 5′-UTR of the FMDV genome initiates viral genome translation in a cap-independent manner [5,6].

Apigenin inhibited FMDV infection at the post-entry stage (Figure 2 and Figure 3). An FMDV IRES-mediated GFP expression plasmid was constructed to explore the hypothesis that apigenin inhibits viral IRES-mediated translation to restrict FMDV infection. As shown in Figure 4A, an FMDV IRES element was inserted into the upstream of pEGFP-C1. This element subjected the expression of GFP under the control of FMDV IRES but in a cap-independent manner. Apigenin exerted no significant inhibitory effect on the expression of GFP when GFP translation was initiated in a cap-dependent manner (Figure 4B). However, GFP expression was significantly suppressed when placed under the control of FMDV IRES in the presence of apigenin in a dose-dependent manner (Figure 4C). Semi-quantitative PCR assay was performed to investigate whether or not apigenin affects the transcription of IRES-GFP. As shown in Figure 4D, the cDNA level of GFP was not significantly affected by apigenin even at high concentrations. These results suggest that apigenin did not influence the transcription of IRES-GFP but restricted IRES-mediated translational activity. Moreover, apigenin targeted the FMDV IRES element to restrict viral infection.

In conclusion, apigenin inhibited FMDV infection by suppressing IRES-driven translational activity. Therefore, apigenin may be a potential small molecular drug to restrict FMDV infection. However, the further research should focus on the use of apigenin *in vivo*.

## Supplementary Files

Supplementary File 1
