# Supplementary material for: Apigenin Restricts FMDV Infection and Inhibits Viral IRES Driven Translational Activity
Source: Viruses. 2015 Mar 31;7(4):1613–26. doi: 10.3390/v7041613 (PMC4411668; doi:10.3390/v7041613)
Supplement: Supplementary File 1 [file viruses-07-01613-s001.pdf]

Supplementary Information

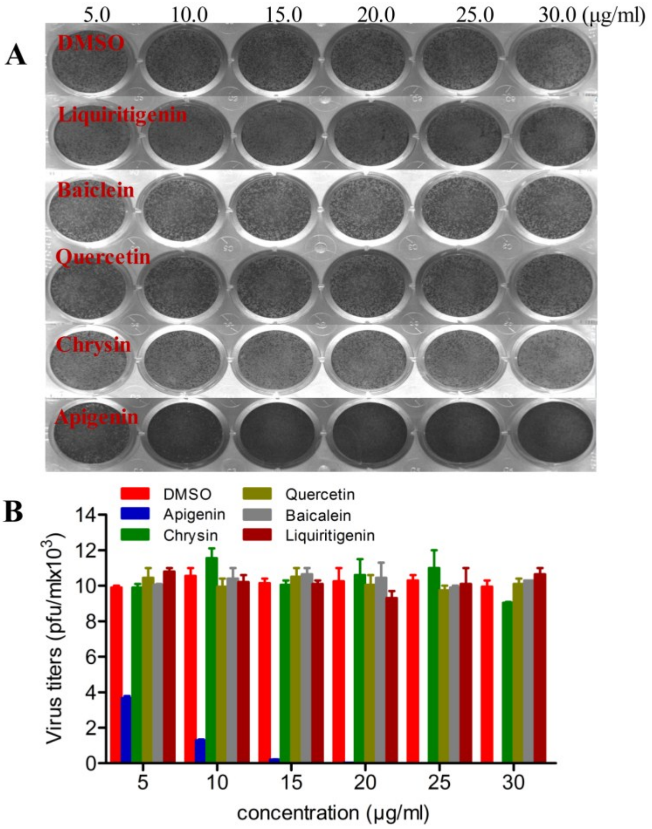

Figure S1. Apigenin inhibits FMDV infection in BHK-21 cells. BHK-21 cells were seeded in 96-well plate and infected with FMDV at a MOI of 0.1. Cells-infected with FMDV were treated with or without different flavonoid at indicated concentration for 24 h. The progeny virion production was determined using PFU assay in BHK-21 cell. Cells were overlaid with 2% methylcellulose. Plaques were fixed with 10% formaldehyde and visualized with crystal violet staining 48 h post infection (**A**); Virus titers determination (**B**).

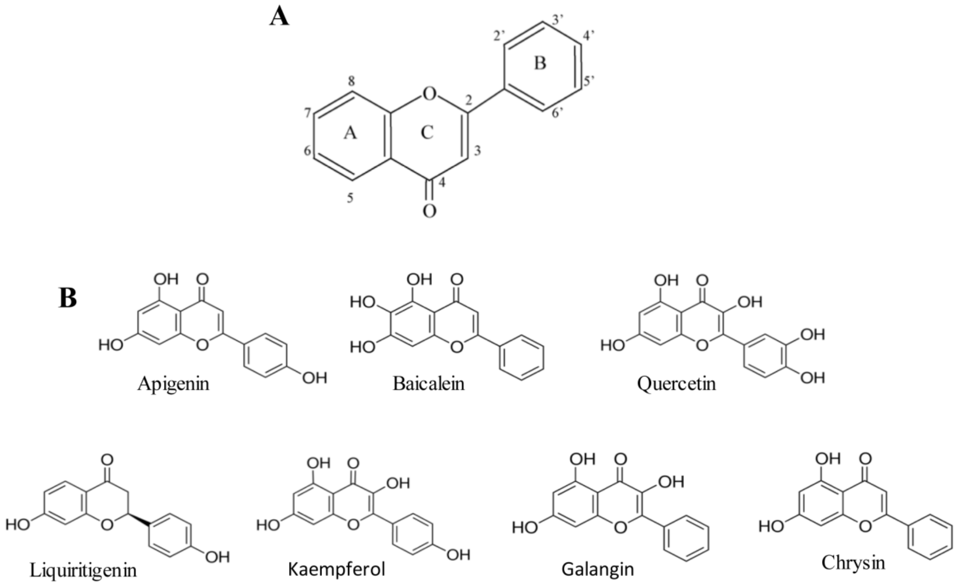

Figure S2. Structural formula of seven flavonoids.
